# The relationship between family intimacy and relapse tendency among people who use drugs: a moderated mediation model

**DOI:** 10.1186/s13011-021-00386-7

**Published:** 2021-06-08

**Authors:** Xiaoqing Zeng, Mengyao Lu, Meirong Chen

**Affiliations:** 1 School of Psychology, Institute of Psychology, Jiangxi Normal University, Nanchang, China; 2 Department of Education, Nanchang Normal University, Nanchang, China

**Keywords:** Family intimacy, Relapse tendency, Positive psychological capital, Self-efficacy

## Abstract

**Background:**

Drug addiction is difficult to overcome. The relapse rate is high, and the negative impact on individuals, families and society is severe, therefore exploring social psychological mechanisms to reduce relapse has very important theoretical and practical value. However, the underlying mechanism by which the interaction between family and individual factors influences the tendency to relapse remain unclear. Thus, the purpose of this paper is to discuss the relationship between family intimacy and relapse tendency of people who use drugs, as well as the mediating effect of psychological capital and the role of self-efficacy in it.

**Methods:**

A total of 817 male who use drugs were investigated via the Family Intimacy and Adaptability Scale, General Self-Efficacy Scale, Positive Psychological Capital Questionnaire and Relapse Tendency Questionnaire. Using Hayes’s process macro carried out moderated mediation analysis.

**Results:**

(1) The average family intimacy score of people who use drugs was low. (2) Family intimacy negatively predicted relapse tendency in people who use drugs. (3) Psychological capital mediated the relationship between family intimacy and relapse tendency. (4) The first half of the indirect effect of family intimacy on relapse tendency was regulated by self-efficacy, compared with the low level of self-efficacy, the psychological capital level with high self-efficacy is higher.

**Conclusion:**

The results of this study suggest that the intimacy between the people who use drugs and their family members should be improved, and the rehabilitation center should take various measures to enhance the psychological capital level and the level of self-efficacy of the people who use drugs, which will be helpful to reduce their relapse tendency.

## Background

More than 35 million people worldwide are currently addicted to drugs, and COVID-19 has created new routes for drug trafficking that increase the risk of drug addiction, making it vital to address this problem [1]. During the COVID-19 pandemic, rehabilitation centres strictly abide by epidemic prevention measures, prohibiting external personnel, including family members, from contacting the people who use drugs (PWUD). The aim is to effectively protect the physical health of PWUD and prevent the combination of COVID-19 and the physiological and psychological effects of drugs from affecting the relapse behaviour of PWUD [2]. Isolation due to COVID-19 may have some impact on family functioning and family intimacy; however, the combined effect on the psychological (anxiety), social (social support, family emotional connection) and behavioural (substance abuse, relapse) of PWUD in complete isolation is currently unknown. Little is known about the psychosocial processes in PWUD, and the theoretical basis of relapse research is still being developed [3, 4]. Participants in this study were all PWUD in compulsory isolation and rehabilitation institutions. PWUD cannot be exposed to drugs during drug rehabilitation. Therefore, relapse tendency can be used as a predictor of relapse behaviour after drug rehabilitation [5]. The purpose of this study was to investigate the relapse tendency of Chinese PWUD in compulsory isolation.

### The relationship between family intimacy and relapse tendency

The family is the smallest unit in a person’s social group. The functioning of the family can influence the formation of an individual’s character, values and social adaptability [6, 7]. Family functioning predicts the number of drugs used by individuals [8], the propensity to use drugs [9] and relapse tendency [10]. Family intimacy is one of the dimensions of family functioning and reflects the cohesiveness of emotional ties between family members [11]. It has a strong relationship with substance addictive behaviour [12]. Several studies have shown that closeness among family members is strongly related to relapse in substance abuse; for example, family intimacy negatively predicts the occurrence of drug use problems in individuals [13] while having a dampening effect on alcohol and drug use [14]. High levels of family intimacy can have a protective, stabilizing effect on drug and alcohol problems [8, 15], helping individuals cope positively with negative events, increasing psychological repair ability [16], and reducing the occurrence of negative events such as drug use to escape stress [17]. However, poor family emotional connections can increase the risk of substance use [18]. Social support theory [19] suggests that family support is an important component of social support, and individuals with benign family support have higher levels of family functioning [20], enabling individuals to positively cope with and mitigate negative emotions and stress.

### The mediating role of psychological capital

Although family intimacy is an important external factor influencing relapse among PWUD, how family intimacy affects the occurrence of relapse behaviour needs to be further explored. This study found, based on a literature review, that the most important cause of relapse may be negative psychological qualities caused by family influence, such as lack of goals and hope in life, negative approach to life events, difficulty recovering from failure [21, 22]. Consequently, this study introduces psychological capital to explore its mediating effect on the relation between family intimacy and relapse tendency among PWUD receiving compulsory treatment.

Positive qualities are core elements on which individuals depend for survival and development and are effective for inhibiting the occurrence of behavioural problems [23]. Positive psychological capital is a positive psychological state that individuals exhibit during development and is manifested in four areas: self-efficacy, resilience, hope, and optimism [24]. Several studies in recent years have found that family functioning has an important effect on core components of psychological capital, such as self-efficacy [25] and resilience [26]. The family functioning model [27] suggests that the basic functions of the family provide certain environmental conditions for the healthy development of family members physically and psychosocially and that good family functioning promotes individuals’ positive emotional involvement and emotional responses and fosters their positive psychological qualities. Similarly, family intimacy, a measure of family functioning, promotes good communication and problem-solving skills among family members [28] and is able to promote the development of their positive psychological qualities [29]. It has been found that individuals with high family intimacy have significantly higher self-efficacy than those with low family intimacy [30] and are more hopeful and confident about the future and more likely to believe in their ability to refuse drugs [31]. However, poor family intimacy can trigger negative emotions such as low self-esteem and depression [30] and difficulties in emotion regulation, hindering the development of positive psychological qualities [32]. Thus, it is evident that close family emotional ties can enhance the positive psychological capital of PWUD, i.e., enhance their optimism, self-confidence, resilience, self-efficacy and other positive psychological qualities.

Psychological capital significantly increases individual motivation and proactive and optimistic responses to crisis events and reduces negative emotions and behaviours [33]. Negative psychological qualities and emotions are prevalent in the addiction treatment population, especially the negative emotional responses that arise after withdrawal, which have a significant impact on relapse [34]. The broaden-and-build theory of positive emotions [35] suggests that positive emotions can expand people’s thought-action systems, help them develop new problem-solving strategies, and dissipate the effects of the narrowed thought-action repertoire produced by negative emotions. Thus, high levels of psychological capital in PWUD can effectively regulate individuals’ negative emotions and problem behaviours [36]. High levels of positive psychological qualities can help individuals quickly regulate stress from external stimuli and develop good social adjustment [37] and can prevent relapse behaviour [38]. Therefore, it can be hypothesized that psychological capital can predict relapse behaviour in PWUD.

### The moderating role of self-efficacy

Self-efficacy influences not only individual behaviour [39] but also individual psychological traits [40], and it thus has important moderating effects on individual psychological traits and behaviour. Psychological capital as a psychological trait is naturally influenced by the regulatory effect of self-efficacy. Self-efficacy is the belief that an individual can successfully perform a specific task or behaviour [41], and it can enhance an individual’s confidence in his or her ability to obtain desired outcomes and develop positive emotions [42]. It has been found that individuals with high self-efficacy have a strong belief in their ability to accomplish their goals, and this inspires them to have hope in life [8], adopt an optimistic approach to external stimuli [43], and rely on positive qualities such as resilience in difficult situations [44]. High self-efficacy also inhibits the emergence of negative emotions and problem behaviours [45] and promotes the maintenance of a positive mental state [46]. Some studies have also found that self-efficacy is an effective moderator of daily life stress and mental health [47]. PWUD with low self-efficacy often show inaction or a lack of determination and perseverance to overcome difficulties and are more likely to experience “learned helplessness” [48], to deal with problems in a negative frame of mind and to be prone to negative emotions such as anxiety, which affects their overall level of psychological capital [49]. In other words, self-efficacy is a strong predictor of individual coping style, performance level and positive belief when facing difficult problems [50]. Compared with individuals with high levels of family intimacy, individuals with low levels of family intimacy are more likely to have negative emotions, feel pessimistic about life, have a strong sense of inferiority, and lack confidence [51, 52]; individuals with a high level of self-efficacy, which is lacking in those with a low level of family intimacy, have firm confidence, a high sense of achievement and weaker feelings of inferiority [53, 54]. This means that close family affective connections alone are not enough to develop individuals’ positive psychological capital, and it is of great significance to investigate the interaction between self-efficacy and family intimacy.

**Fig. 1:**
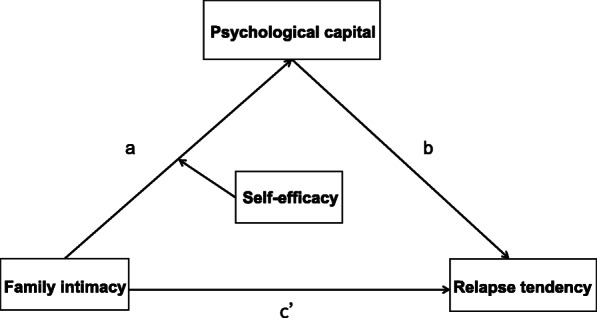
A moderated mediating model

In short, these variables rarely appear in isolation; rather, they appear in groups such that they attract and form building blocks with each other. However, the interaction between family factors and personal factors and the underlying mechanisms remain unclear. Mediation and moderation analyses can be used to determine the relationships between these variables, thus making it a worthy topic for further study. In addition, existing research has mainly focused on persons undergoing voluntary detoxification and community drug treatment in Western countries. Thus, it is necessary to perform research on PWUD undergoing compulsory isolation in an Asian context. Finally, studying the joint effects of these variables is the key to understanding the basis of drug addiction and developing effective treatment methods as a next step. Even more important is determining the supportive psychological factors in PWUD to provide new ideas for helping such persons more successfully remain abstinent in the long term. We constructed a moderated mediation model (see Fig. 1). Therefore, we propose Hypothesis 1: The family intimacy of PWUD negatively predicts their relapse tendency; Hypothesis 2: The psychological capital of PWUD plays a mediating role in the relationship between family intimacy and relapse. Hypothesis 3: Self-efficacy plays a moderating role in the relationship between family intimacy and psychological capital.

## Methods

### Participants

Cluster random sampling method was used to select more than 1800 PWUD from a large drug rehabilitation center in Jiangxi Province, China from May to August 2020. First of all, PWUD has been divided into different groups in the drug rehabilitation center, it is generally divided into 20 groups according to the drug rehabilitation center. Secondly, a simple random method was adopted to select 10 groups out of 20 groups. Individuals with poor physical conditions and those who were difficult to participate in the rehabilitation center were excluded. All the individuals in the selected groups were included in the survey. A total of 880 PWUD were investigated. After eliminating 63 PWUD with incomplete answers, 817 valid questionnaires were left, with an effective response rate of 92.8%.

### Measures

#### Demographic characteristics

Background data were recorded for each participant, including age, educational level (elementary school or below, junior high school, senior high school or technical secondary school, junior college, undergraduate degree or above), marital status (unmarried, married, remarried, divorced or widowed), place of residence (urban areas, rural areas), and type of drug use (meth, ketamine, opium, heroin, others).

#### Family intimacy and adaptability scale

We used the Family Intimacy and Adaptability Scale [28]. The original scale consists of two scales measuring respondents’ actual feelings about the current state of their family and the ideal family situation they would like to have, with 30 items each. The subscale of actual feelings about one’s family situation includes two factors: intimacy and adaptability. Intimacy refers to the perceived emotional connection among family members (e.g., “Family members do their best to support each other when there are difficult times”). A 5-point scale was used (1 = “not”, 5 = “always”). Only participants’ intimacy scores were counted in this study; higher scores indicated higher levels of family intimacy, and Cronbach’s α was .89 in this study.

#### General self-efficacy scale

The General Self-Efficacy Scale is unidimensional [55], with a total of ten items (e.g., “I’m confident that I can cope effectively with anything that comes up unexpectedly”) rated using a 4-point scale (1 = “not correct”, 4 = “correct”). Higher scores indicate higher general self-efficacy, and Cronbach’s α was .84 in this study.

#### Relapse tendency questionnaire

We used the Relapse Tendency Questionnaire [56], which has 18 items measuring the willingness to quit drugs (“I will encounter many difficulties and setbacks in the process of drug rehabilitation, and I am fully prepared for this”), substance substitution (“I hope to have the opportunity to enjoy drugs again and enjoy my addiction”), objective circumstances (“After I get clean, I want to move to a new place where no one knows me so that I can get rid of my drug friends and drugs completely”). The five dimensions were physical and mental condition (“physical condition after long-term drug use”) and social support (“encouragement and support from family, relatives, and friends for me to quit drugs”). A 6-point scale was used (0 indicating the least severe degree and 5 indicating the most severe degree), and a higher total score indicated a higher relapse tendency. Cronbach’s α was .91 in this study.

#### Positive psychological capital questionnaire

We used the Positive Psychological Capital Questionnaire [57]. It assesses self-efficacy (“I am confident in my abilities”), resilience (“Bad experiences can depress me for a long time”), hope (“I am working hard to achieve my goals “) and optimism (“I tend to hang my head when things don’t go my way”) in four dimensions, with 26 entries in total, using a 7-point scale (1=“not at all consistent”, 7 = “completely consistent”). Higher scores indicate higher levels of positive psychology in each dimension or overall. Cronbach’s α was .87 in this study.

### Administration procedures

The test was administered by a trained graduate student in psychology. Before the test, the tester read out the instructions and explained that the test was for scientific research only, that the questionnaire should be filled out according to the respondents’ actual circumstances, that participants could withdraw or discontinue answering the questionnaire at any time, and that the confidentiality of the test results was guaranteed. All participants first signed the informed consent form and then completed the questionnaire. After completing the survey, each questionnaire was collected on-site, and participants received a gift worth $3. The entire process was completed in a spacious, quiet, and air-conditioned classroom and lasted approximately 30 min. All participants were tested by the rehabilitation hospital and were free of mental illness according to the DSM-IV criteria.

### Data analysis

First, the common method deviation test was carried out. Using AMOS 24.0, family intimacy, self-efficacy, positive psychological capital, and relapse tendency were fixed as one factor, and the number of common factors was set as 1 for confirmatory factor analysis to check the model fit based on the hypothesis that “a single factor explains all the variation”. Second, descriptive tests, difference analyses and correlation analyses were conducted on the results. SPSS 23.0 was used to conduct Spearman grade correlation for the four variables of family intimacy, self-efficacy, positive psychological capital and relapse tendency, and independent sample t-tests and one-way analysis of variance (ANOVA) were used to test whether there were significant differences in relapse tendency among respondents with different demographic characteristics. Third, this study examined the mediating role of psychological capital between family intimacy and relapse tendency via mediation analysis [58]. In addition, we estimated the mediation proportion: how large a proportion of the total effect of family intimacy on relapse tendency is mediated through psychological capital. The algorithm used was: mediation proportion = (ab)/c [59, 60]. Fourth, this study examined whether the first half of the mediation process was moderated by self-efficacy. Moderated mediation is often used to test whether the size of the mediation effect depends on the value of the moderating variable [61]. Moderated mediation analysis was carried out using Hayes’s process macro (Model 7). In addition, the bootstrap method was used to test the significance of all effects to obtain the standard error of parameter estimation [62]. The bootstrap method generates 95% bias-corrected confidence intervals for these effects from 5000 resampled datapoints, and confidence intervals that do not include zero indicate significant influence.

## Results

### Common method bias

Because this study used self-reporting methods to collect data, common method bias may exist. To reduce the impact of common method bias on the study results, we used procedural controls and statistical method test controls. For the procedural control, we adopted a questionnaire design with a randomized order of questions, and for the statistical control, we tested the common method bias by structural equation modelling using a common factor of 1 for the validation factor analysis to test the hypothesis that “a single factor explains all the variation” more precisely [63]. Using Amos 24.0, family intimacy, self-efficacy, positive psychological capital, and relapse tendency were fixed as one factor to check the model fit: *χ*^*2*^ = 30,060.969 (*p* < 0.001), *df* = 3486, *χ*^*2*^*/df* = 8.623, *CFI* = 0.000, *TLI* = 0.000, *RMSEA* = 0.097, *SRMR* = 0.166. The model fit was poor, indicating that there was no serious common method bias in this study.

### Descriptive statistical analysis

Table 1 shows the sociodemographic characteristics. A total of 817 participants were included in the study. The participants’ ranged in age from 17 to 62 years, with a mean age of 34.45 ± 8.28 years.

**Table 1:** Characteristics of PWUD(*N* = 817)

Variables		*N*	Percent
Educational level	elementary school or below	131	16.0
	junior high school	466	57.0
	senior high school or technical secondary school	188	23.0
	junior college	23	2.9
	undergraduate degree or above	9	1.1
Marital status	unmarried	273	33.4
	married	306	37.5
	remarried	32	3.9
	divorced or widowed	206	25.2
Place of residence	urban areas	496	60.7
	rural areas	321	39.3
Types of drug use	meth	609	74.5
	ketamine	105	12.9
	opium	2	0.2
	heroin	83	10.2
	other	18	2.2

### Differences in relapse tendency among groups differing by marital status, education level, and type of drug used

To investigate whether there were differences in relapse tendency by age, marital status, education level, place of residence, and type of drug used, these variables were used as grouping variables, and independent sample t-tests or one-way ANOVA was conducted. The results are shown in Table 2. The *LSD* post hoc test showed that PWUD who were “remarried”(32.12 ± 16.04) were more likely to relapse than those who were “unmarried”(27.45 ± 13.06), “married”(25.72 ± 15.01) and “divorced or widowed”(26,94 ± 14.20). PWUD with “ elementary school or below”(31.02 ± 15.97) score higher than those with “junior high school”(25.98 ± 13.77), “ senior high school or technical secondary school “(26.05 ± 13.67); PWUD who used “heroin”(31.01 ± 14.18) and “other drugs”(35.30 ± 18.74) scored higher than those who used “meth”(26.13 ± 14.03) and “ketamine”(26.38 ± 13.64). Age (*F* = 1.16, *p* > 0.05) and place of residence (*F* = 0.44, *p* > 0.05) were not associated with statistically significant differences in relapse tendency, and the results are shown in Table 2.

**Table 2:** Marital status, educational level, and types of drug use differ in relapse tendency

		Sum of Squares	Mean square	*F*	*p*
Marital status	Among groups	2275.53	568.88	2.85	0.023
	Within-group	162,276.09	199.85		
	Total	164,551.62			
Educational level	Among groups	3223.394	644.68	3.24	0.007
	Within-group	161,328.22	198.93		
	Total	164,551.62			
Types of drug use	Among groups	3638.20	727.64	3.67	0.003
	Within-group	160,913.42	198.41		
	Total	164,551.62			

### Descriptive statistics and correlation analysis

Table 3 lists the mean, standard deviation and correlation analysis results for each variable. Compared with the Chinese norm (63.9 ± 8.0), the family intimacy score of PWUD (62.35 ± 10.64) was low (*p* < 0.01). The results of the correlation analysis showed that family intimacy, psychological capital and self-efficacy were significantly negatively correlated with relapse tendency. There was a significant positive correlation between family intimacy, psychological capital and self-efficacy and a significant positive correlation between family intimacy and psychological capital.

**Table 3:** Means, Standard Deviations (*SD*), and Correlations of all variables

	*M*	*SD*	1	2	3	4
1.Family intimacy	62.35	10.64	1.00			
2.General self-efficacy	2.47	0.55	.244^**^	1.00		
3.Relapse tendency	26.85	14.20	−.150^**^	−.155^**^	1.00	
4.Psychological capital	4.46	0.74	.367^**^	.479^**^	−.285^**^	1.00

^**^ *p* < 0.01

### The relationship between family intimacy and relapse tendency: a moderated mediation model test

First, relapse behaviour differed significantly by participants’ marital status, education level, and type of drug use. Therefore, the mediating effect of psychological capital on the relationship between family intimacy and relapse tendency was tested using Model 4 (Model 4 is a simple mediation model) in the SPSS macro developed by Hayes [62], controlling for the variables of marital status, education level, and type of drug use. The results (see Table 4) showed that the negative predictive effect of family intimacy on relapse tendency was significant (*β* = − 0.19, *t* = − 4.13, *p* < 0.001; “*β*” is the standardized coefficient), but when the mediating variable was entered, the predictive effect of family intimacy on relapse tendency was not significant (*β* = − 0.07, *t* = − 1.44, *p* > 0.05). Therefore, Hypothesis 1 was verified. The positive predictive effect of family intimacy on psychological capital was significant (*β* = 0.02, *t* = 10.79, *p* < 0.001), as was the negative predictive effect of psychological capital on relapse tendency (*β* = − 5.01, *t* = − 7.17, *p* < 0.001).

**Table 4:** The mediation model test of psychological capital

Outcome variables	Independence variables	*R*^*2*^	*F*	*β*	*t*
Relapse tendency		0.04	9.12^***^		
	Marital status			0.13	0.31
	educational level			−1.22	−1.85
	types of drug use			1.31	3.70^***^
	Family intimacy			−0.19	−4.13^***^
Psychological capital		0.16	38.44^***^		
	Marital status			−0.002	−0.11
	educational level			0.16	4.86^***^
	types of drug use			−0.005	−0.26
	Family intimacy			0.02	10.79^***^
Relapse tendency		0.10	18.02^***^		
	Marital status			0.12	0.29
	educational level			−0.44	−0.68
	types of drug use			1.29	3.75^***^
	Family intimacy			−0.07	−1.44
	Psychological capital			−5.01	−7.17^***^

^***^ *p* < 0.001

The 95% bootstrap confidence interval for the direct effect of family intimacy on relapse tendency and the mediating effect of psychologi

cal capital did not contain 0 (see Table 5), indicating that family intimacy not only directly predicted relapse tendency but also predicted relapse behaviour through the mediating effect of psychological capital; thus, Hypothesis 2 was verified. This direct effect (− 0.07) and mediating effect (− 0.10) accounted for 41.18 and 58.82% of the total effect (− 0.17), respectively.

**Table 5:** Mediating effect analysis of psychological capital

	Standardization effect estimation	Relative effect value	The Boot Standard Error (SE)	95% Confidence interval (CI)
The total effect (ab+c’)	− 0.10 + (− 0.07) = − 0.17	100%	0.05	−0.28, − 0.10
Direct effect(c’)	− 0.07	41.18%	0.05	− 0.16, 0.03
Mediating effect (ab)	0.02 × (− 5.01) = − 0.10	58.82%	0.02	−0.17, − 0.08

**Fig. 2:**
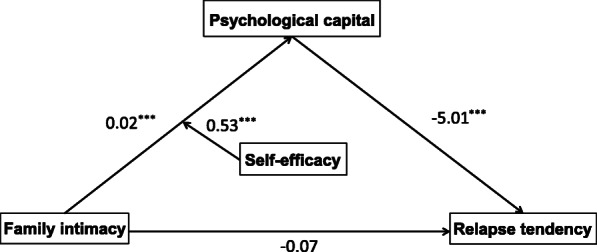
Path coefficients of family intimacy, self-efficacy, pyschological capital and relapse tendency. ***p < 0.001

Second, the moderated mediation model was tested using Model 7 of the *SPSS* macro developed by Hayes (Model 7 assumes that the first half of the mediation model and the direct path are moderated, consistent with the theoretical model of this study), controlling for the variables of marital status, education level, and type of drug use. The results showed (see Table 6) that there was a significant effect of family intimacy on psychological capital after general self-efficacy was entered into the model (*β* = 0.02, *t* = 8.62, *p* < 0.001), and the product term of family intimacy and general self-efficacy was a significant predictor of psychological capital (*β* = 0.01, *t* = 2.97, *p* < 0.001), indicating that general self-efficacy was able to moderate the predictive effect of family intimacy on psychological capital. The detailed path model is shown in Fig. 2.

**Table 6:** Moderated mediation model testing

Outcome variables	Independence variables	*R*^*2*^	*F*	*β*	*t*
Psychological capital		0.32	62.81^***^		
	Marital status			0.01	0.33
	educational level			0.12	3.98^***^
	types of drug use			0.01	0.70
	Family intimacy			0.02	8.62^***^
	General self-efficacy			0.53	13.21^***^
	Family intimacy × General self-efficacy			0.01	2.97^***^
Relapse tendency		0.10	18.02		
	Marital status			0.12	0.29
	educational level			−0.44	−0.68
	types of drug use			1.29	3.75^***^
	Family intimacy			−0.07	−1.44
	Psychological capital			−5.01	−7.17^***^

^***^ *p* < 0.001

**Fig. 3:**
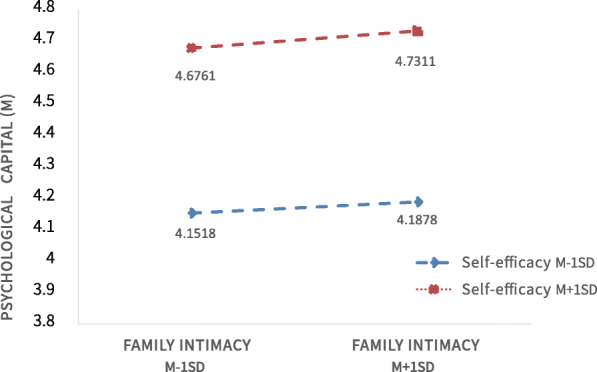
Moderating effect of general self-efficacy on family intimacy with psychological capital

To understand the nature of the interaction effect between family intimacy and self-efficacy, participants with different levels of self-efficacy were analysed separately, and the mediated effect values and 95% bootstrap confidence intervals for psychological capital between family intimacy and relapse tendency for both groups of participants are shown in Table 7 (“*Slope*” in the table is a standardized slope estimate). Further simple slope analysis showed (see Fig. 3) that for participants with higher levels of self-efficacy (*M* + 1*SD*), family intimacy had a significant positive predictive effect on psychological capital (*simple slope* = 0. 023, *t* = 8.32, *p* < 0. 001), whereas for participants with lower levels of self-efficacy (*M*-1*SD*), family intimacy also had a positive predictive effect on psychological capital (*simple slope* = 0. 013, *t* = 4.78, *p* < 0. 001). The predictive effect was small, indicating that the predictive effect of family intimacy on psychological capital gradually increased as the level of self-efficacy increased (see Table 6). Therefore, Hypothesis 3 was verified.

**Table 7:** The mediating effect of psychological capital on different levels of self-efficacy

Self-efficacy level	*Slope*	Bootstrap Standard Error	LLCI	ULCI
M-1SD	−0.06	0.02	−0.10	− 0.03
M ± 1SD	−0.09	0.02	−0.13	− 0.06
M + 1SD	−0.12	0.02	−0.17	− 0.07

*“Slope”*is a standardized slope estimate

## Discussion

### The relationship between family intimacy and relapse tendency in PWUD

In this study, although there is a small correlation between family intimacy and relapse tendency, family intimacy was still a negative predictor of relapse tendency, which is consistent with previous studies [12]. From the point of view of PWUD, during the COVID-19 pandemic, due to the necessity of implementing prevention measures to mitigate disease transmission, drug rehabilitation centres temporarily forbade visits, communication between PWUD and their families was temporarily blocked, and the emotional connection with family was weakened [64],these may be different from other studies showing a high negative correlation between family intimacy and relapse tendency [13]. In addition, the traditional service industry and manufacturing industry are under great threat [65], and there have been layoffs, wage arrears, bankruptcy and other phenomena that have aggravated the psychological distress of PWUD with respect to the economic situation of their families [66], making them prone to negative emotions. Moreover, compared to people in individualistic cultures, Chinese people, who live in a collectivist culture, are more introverted, and most individuals are reluctant to express their emotions openly and tell their loved ones about stressful events; instead, they generally choose to hide their emotions [3]. On the other hand, drug use is a crime in China, and it is often not tolerated even within the family; families may minimize mention of and avoid talking about the PWUD due to the traditional culture of “not washing your dirty linen in public” [67]. This reduces the PWUD’s feelings of emotional closeness to the family, i.e., the individual does not have positive experiences of emotional connection with the family, which also explains the low family intimacy scores. From the point of view of the families of PWUD, because recovering PWUD have to deal with the multiple pressures as they readjust and reintegrate into social life after leaving rehab, if PWUD do not receive emotional support and respect from family members, instead experience poorer family intimacy due to stigmatization [31], which may lead to relapse behaviour [68]. Indeed, family members with low family intimacy are more likely to experience negative emotions due to poor communication, increase family conflict, and create a violent or indifferent family atmosphere [69], failing to encourage positive emotions in the recovering PWUD or even to properly guide the PWUD to a hopeful life and enhance ability to recover quickly from adversity [21]. The average age of the participants in this study was 34.41 ± 8.01 years, which is the age at which Chinese begin to formally assume more responsibilities in their families of origin and nuclear families and at work, but the participants current drug use behaviours do not match the roles and responsibilities they should be assuming. Self-difference theory [70] suggests that there may be a discrepancy between an individual’s “real self” and “ought self”. That is, an individual may believe that his current drug-taking behaviour is inconsistent with the behaviours that important others (such as parents, children, and lovers) think an individual should engage in to fulfil his responsibilities and obligations, and this difference leads to the generation of negative emotions. The negative reinforcement emotion processing model [71] suggests that avoidance of negative emotions is the preferred motivation for individuals to maintain addictive behaviours and that PWUD may resort to additional substance abuse behaviours to avoid negative emotions, which may further trigger relapse behaviours. Therefore, in China, the family members of PWUD should be encouraged to cooperate with each other and with the rehabilitation centre and participate in the rehabilitation process. In terms of communication skills, in special cases, the drug rehabilitation centre should take advantage of existing technology, such as “cloud” communication, to enhance the emotional connection between family members and PWUD.

### Analysis of the mediating role of psychological capital in the relationship between family intimacy and relapse tendency

This study found that psychological capital played a mediating role in the relationship between family intimacy and relapse tendency. This is consistent with previous studies [72]. Good communication and strong emotional ties among family members are predictors of the development of positive qualities such as optimism and self-efficacy [29], whereas family conflict and low levels of family intimacy predispose individuals to problems such as pessimism and difficulties with social adjustment [73] and a lack of good coping strategies. High levels of psychological capital are strongly associated with less substance abuse and relapse behaviour [74], and the Hope Therapy educational intervention study showed that positive psychotherapy was effective in reducing depression and predicting relapse in people who sought addiction treatment [75]. Conversely, individuals with less psychological capital are more impulsive, helpless, and prone to reliance on substance-abusing behaviours to regulate their emotions and escape stress, making PWUD prone to relapse [34].

Triadic reciprocal determinism (TRD) [76] states that individuals’ subjective factors, the environment they live in, and the behaviours they exhibit are interactively determined. That is, the emergence of relapse behaviours in recovering PWUD is influenced not only by the family environment but also by their traits such as their beliefs, cognition, and psychological capital [10]. Thus, even when recovering PWUD feel the same level of family emotional connection, differences in psychological capital can influence hope and optimism about future lives, PWUD’s belief that they can quit drugs, and their ability to make behavioural choices related to substance abuse. However, it has also been shown that levels of psychological capital are influenced by family [72]. PWUD who perceive good family emotional connections have higher self-confidence and positive optimism and possess the determination and hope to rebuild their lives and reform, and receiving emotional support from family enables individuals to recover quickly from difficult situations and reduce use of drugs to de-stress. When a recovering PWUD perceives poor emotional relationships among family members, on the one hand, the individual is affected by stigmatization, and the indifferent and poor attitudes of family members make the individual believe that everyone rejects him or her. On the other hand, the family is the most important and influential source of social support for the individual, but the recovering PWUD does not receive emotional support from family. This can lead to a loss of hope in life and confidence in recovery, making it difficult to resolve negative emotions and eventually leading to re-engagement in drug use [77]. Therefore, psychological capital also contributes to the reduction of relapse tendencies, and rehabilitation centres can conduct group counselling activities that enhance positive psychological qualities such as optimism, self-efficacy, resilience, and hope, which can help PWUD recover quickly and positively from imbalances caused by stressful events and reduce substance abuse cravings and relapse behaviours [78].

### Analysis of the moderating role of self-efficacy

The results of this study indicate that the effect of family intimacy on the psychological capital of PWUD is moderated by self-efficacy, and there are differences in the effect of family intimacy on psychological capital at different levels of self-efficacy. When the level of self-efficacy of PWUD is low, the predictive effect of family intimacy on psychological capital is small; conversely, when the level of self-efficacy of PWUD is high, the predictive effect of family intimacy on psychological capital is large. This result is consistent with previous studies on the protective role of self-efficacy [79]. Fergus and Zimmerman [80] proposed that the protective factors that prevent negative effects may be positive factors internal to the individual (e.g., competence, coping skills, and self-efficacy) or positive factors external to the individual (e.g., parental support, adult guidance). The protective-stability model [15] proposes that protective factors help to counteract the effects of risk, with higher levels of risk being associated with higher levels of negative outcomes when protective factors are absent but no relationship between risk and outcome existing when protective factors are present. That is, both family intimacy and self-efficacy are protective factors that can have a reinforcing effect on the improvement of psychological capital levels, i.e., high levels of self-efficacy can both promote higher levels of psychological capital in recovering PWUD who have good family intimacy and mitigate the negative effects of low family intimacy and improve psychological capital levels. Low family intimacy means that recovering PWUD have difficulty obtaining effective emotional support from their families and do not perceive close emotional connections among family members; thus, the negative emotions cannot be confided in their families in a timely manner, which can trigger negative psychological emotions such as anxiety and depression, leading to a decrease in forms of psychological capital such as optimism and hope [81]. Conversely, PWUD with high self-efficacy have more strategies to mitigate and cope with the effects of various negative emotions (such as cognitive reappraisal) [82], and PWUD can buffer the negative effects of family conflict, use positive reframing strategies to cope with challenges, focus on the optimistic aspects of the situation, and quickly recover from failure and be hopeful about new goals for the future [8]. Therefore, rehabilitation centres should carry out physical rehabilitation training, such as Qigong, Taijiquan, yoga, and free rotation aerobic exercise [83]. From the perspective of external intervention, the symptoms of depression and negative emotions caused by decreased dopamine release can be reduced to improve the success rate of withdrawal and reduce drug craving and relapse. Embodied cognition theory suggests that cognitive processes are influenced by both the body and the environment and that there is a strong link between physical experiences and psychological states [84]. When the body is restored, individuals can feel positive and happy emotions, which can improve their self-confidence and self-efficacy.

### Limitations

The following limitations and shortcomings remain in this study. First, this study used a cross-sectional design, and although we adopted procedural and statistical controls to minimize the effects of common method bias, as with all cross-sectional studies, a longitudinal study is still needed to determine the causal relationships among the variables. Second, the COVID-19 epidemic has affected the psychological stress response and psychological well-being of individuals and family members, which can lead to abnormal behaviours related to drug used [85]. This study examined the relationship between family intimacy and relapse tendency in PWUD only during the COVID-19 outbreak. For this reason, subsequent studies should consider comparing the relationship between family intimacy and relapse tendency in PWUD found herein with that in nonspecial periods to confirm the influence of the unique family conditions created by the COVID-19 pandemic on relapse tendency. Third, this study used questionnaire method, which may have self-report bias. In the follow-up study, in addition to self-assessment, other assessment (for example: family, rehabilitation centers staff) and interview method will be added to make the research results more objective. Fourth, the participants of this study were from male who use drugs in compulsory rehabilitation centers of China. Due to the violation of social order and legal system, the public has generated wrong ideas and negative stereotypes about PWUD. As a result, it is difficult for PWUD to integrate into the society. Therefore, the applicability of this result to voluntary PWUD and female PWUD should be cautious. Finally, according to Chinese law, PWUD needs to take different measures for drug rehabilitation, such as compulsory drug rehabilitation center, community drug rehabilitation, etc., so our findings do not apply to in other societies people who voluntarily stopping drugs or who have a shared history of drug use by family members.

## Conclusion

This study drew the following conclusions: (1) the average family intimacy score of PWUD was low; (2) family intimacy negatively predicted the relapse tendency of PWUD; (3) psychological capital mediated the relationship between family intimacy and relapse tendency; (4) the indirect effect of family intimacy on relapse tendency was moderated by self-efficacy in the first half of the mediation. Compared with PWUD with low self-efficacy, PWUD with high self-efficacy had more psychological capital. Therefore, there are both mediating and moderating effects between family intimacy and relapse tendency. The results of this study suggest that family members of PWUD and rehabilitation centres should be encouraged to participate in the rehabilitation process of PWUD, improve the communication skills of family members and improve the psychological capital level of PWUD to reduce the possibility of relapse tendency.

## Data Availability

The raw data supporting these findings can be found in the supplementary file.

## Abbreviations

PWUD: people who use drugs
SD: Standard Deviation
SE: Standard Error
CI: Confidence Interval
